# Analysis of genetic relationships of genotypes of the genus Rosa L. from the collection of Nikita Botanical Gardens using ISSR and IRAP DNA markers

**DOI:** 10.18699/VJ20.639

**Published:** 2020-08

**Authors:** I.I. Suprun, S.A. Plugatar, I.V. Stepanov, T.S. Naumenko

**Affiliations:** North Caucasian Federal Scientific Center of Horticulture, Viticulture, Wine-making, Krasnodar, Russia; Order of the Red Banner of Labour Nikita Botanical Gardens – National Scientific Center of the Russian Academy of Sciences, Yalta, Republic of the Crimea, Russia; North Caucasian Federal Scientific Center of Horticulture, Viticulture, Wine-making, Krasnodar, Russia; Order of the Red Banner of Labour Nikita Botanical Gardens – National Scientific Center of the Russian Academy of Sciences, Yalta, Republic of the Crimea, Russia

**Keywords:** Rosa L., rose, genetic resources, DNA-markers, ISSR, IRAP, genetic diversity, Rosa L., розы, генофонд, генотипирование, ДНК-маркеры, IRAP, ISSR, генетическое разнообразие

## Abstract

In connection with the development of breeding and the creation of new plant varieties, the problem of their genotyping and identification is becoming increasingly important, therefore the use of molecular methods to identify genetic originality and assess plant genetic diversity appears to be relevant. As part of the work performed, informative ISSR and IRAP DNA markers promising for the study of genetic diversity of the Rosa L. genus were sought and applied to analysis of genetic relationships among 26 accessions of the genus Rosa L. from the gene pool collection of Nikita Botanical Gardens. They included 18 cultivated varieties and 8 accessions of wild species. The species sample included representatives of two subgenera, Rosa and Platyrhodon. The subgenus Platyrhodon was represented by one accession of the species R. roxburghii Tratt. Cultivated roses were represented by varieties of garden groups hybrid tea, floribunda, and grandiflora. The tested markers included 32 ISSRs and 13 IRAPs. Five ISSR markers (UBC 824, ASSR29, 3A21, UBC 864, and UBC 843) and three IRAPs (TDK 2R, Сass1, and Сass2) were chosen as the most promising. They were used for genotyping the studied sample of genotypes. In general, they appeared to be suitable for further use in studying the genetic diversity of the genus Rosa L. The numbers of polymorphic fragments ranged from 12 to 31, averaging 19.25 fragments per marker. For markers UBC 864 and UBC 843, unique fingerprints were identified in each accession studied. The genetic relationships of the studied species and varieties of roses analyzed by the UPGMA, PCoA, and Bayesian methods performed on the basis of IRAP and ISSR genotyping are consistent with their taxonomic positions. The genotype of the species R. roxburghii of the subgenus Platyrhodon was determined genetically as the most distant. According to clustering methods, the representative of the species R. bengalensis did not stand out from the group of cultivated varieties. When assessing the level of genetic similarity among the cultivated varieties of garden roses, the most genetically isolated varieties were ‘Flamingo’, ‘Queen Elizabeth’, and ‘Kordes Sondermeldung’; for most of the other varieties, groups of the greatest genetic similarity were identified. This assessment reflects general trends in phylogenetic relationships, both among the studied species of the genus and among cultivated varieties.

## Abstract (ru)

В связи с развитием селекции и появлением новых сортов растений все более важным становится вопрос паспортизации и структуризации генофонда, поэтому использование молекулярно-генетических мето- дов для выявления генетической оригинальности и оценки генетического разнообразия растений представля- ется актуальным. В рамках настоящей работы осуществлены поиск информативных ISSR- и IRAP- ДНК-маркеров, перспективных для изучения генетического разнообразия рода Rosa L., и анализ с их помощью генетических взаимосвязей образцов из генофондовой коллекции роз Никитского ботанического сада. Материалом для ге- нотипирования послужили 26 образцов, 18 из которых являются культурными сортами, а 8 образцов относятся к дикорастущим видам. В выборку видов включены представители двух подродов Rosa и Platyrhodon. Подрод Platyrhodon представлен одним образцом вида R. roxburghii Tratt. Среди культурных роз присутствовали сорта садовых групп: чайно-гибридной, флорибунда и грандифлора. В исследовании было задействовано 32 ISSR- и 13 IRAP-маркеров. После апробации были отобраны как наиболее перспективные пять ISSR-маркеров (UBC 824, ASSR29, 3A21, UBC 864, UBC 843) и три IRAP-маркера (TDK 2R, Сass1, Сass2). Они использовались для генотипиро- вания исследуемой выборки генотипов и в целом перспективны для дальнейшего изучения генетического раз- нообразия рода Rosa L. Количество полиморфных фрагментов варьировало в диапазоне от 12 до 31, в среднем 19.25 фрагмента на маркер. По маркерам UBC 864 и UBC 843 выявлены уникальные фингерпринты для каждого изученного образца. Оценка генетических взаимосвязей изученных видов и сортов роз c использованием ме- тодов UPGMA, PCoA и байесовского анализа, выполненная на основе данных IRAP- и ISSR-генотипирования, со- гласуется с таксономическим положением образцов. Генотип вида R. roxburghii подрода Platyrhodon определен как генетически наиболее отдаленный образец. Представитель вида R. bengalensis методами кластеризации не выделился из группы культурных сортов. При оценке уровня генетического сходства среди культурных сортов садовой розы наиболее генетически обособленными сортами оказались ‘Flamingo’, ‘Queen Elizabeth’, ‘Kordes Sondermeldung’, для остальных сортов были определены группы наибольшего генетического сходства. Данная оценка отражает общие тенденции в филогенетических отношениях как между изученными видами рода, так и между культурными сортами.

## Introduction

According to the Plant List database (www.theplantlist.org), the genus Rosa L. includes 373 recognized species. Sixteen of them occur in the natural flora of the Crimea (Ena, 2012). Currently, the world range of garden roses includes more than 30 thousand varieties. The international classification divides all this diversity into 36 garden groups according to its decorative and biological characteristics (McFarland, 2007). Rose breeding efforts have increased in recent years in Japan, China, India, Canada, and New Zealand (Plugatar et al., 2017). In Russia, breeding work with roses has been successfully carried out in the Nikita Botanical Gardens (NBG) since 1824 (Plugatar, 2016). Roses from the garden groups floribunda, grandiflora, miniature, and hybrid tea have flowering periods from 180 to 200 days a year, depending on the variety, and are the most promising and popular in gardening (Plugatar et al., 2017).

The mobilization and preservation of genetic resources of the entire diversity of rose cultivars and species that participated in their creation is one of the main directions in the creation of new varieties that would meet the requirements of modern decorative floriculture for specific regions of cultivation of this crop (Schanzer, Vagina, 2007; Korkmaz, Dogan, 2018).

The current development of DNA marking methods and their introduction into scientific practice contributes to the improvement of the efficiency of research aimed at clarifying the genetic relationships of varieties at the intra- and interspecies level; study of the genetic structure of collections of gene pools; creation of collections; certification and registration of the existing gene pool. In addition, DNA marking methods can be effectively used to seek donors of genes for breedingvaluable traits, identify duplicate accessions, and resolve disputes when classifying newly received specimens. The use of data at the level of genetic similarity in combination with phenotypic characteristics of varieties in the formation of parent pairs in breeding programs can be promising.

The earliest phylogenetic studies of the genus Rosa L. using molecular genetic markers include the work by Millan et al. (1996). The study used RAPD markers (Random Amplified Polymorphic DNA) to assess polymorphism at the intraspecific and interspecific levels in representatives of various sections of Rosa. Three clusters were established by the UPGMA method: the first cluster included accessions of sections Pimpinellifoliae and Synstylae, the second cluster was formed by sections Chinenses (Indicae) and Gallicanae, and the third was represented by species of sections Cassiorhodon (Cinnamomeae) and Caninae. However, a later work with a significantly broader sample of 109 specimens belonging to 39 species (Atienza et al., 2005) failed to obtain an unambiguous distribution of samples by taxa. These results were not completely consistent with a slightly earlier study by American authors (Jan et al., 1999), who also used RAPD markers to analyze another sample of 119 accessions of 36 Rosa species.

Koopman et al. (2008) used AFLP DNA markers for phylogenetic analysis. By analysis of a series of 92 samples belonging to 46 species of the genus Rosa L., the phylogeny of species within the genus Rosa was reconstructed. Multilocus DNA-markers have been widely used to elucidate genetic relationships, both in gene pool collections and in the study of natural populations of various species of the genus Rosa. Thus, using complex data obtained by ISSR and RAPD analysis, a group of scientists from Turkey analyzed genetic relationships among 27 Rosa species growing in Turkey (Korkmaz, Dogan, 2018). RAPD, ISSR, and SSR markers were used to study the genetic relationship between Taif rose accessions and those collected in Syria and Egypt, including the Damascus rose. The analysis of the degree of genetic similarity based on the results of genotyping revealed the greatest degree of genetic affinity of Taif roses to Damascus roses of Gory variety growing in Syria (El-Assal et al., 2014). Molecular analysis methods, including ISSR markers, were also successfully used in a number of works aimed at studying the genetic relationships in the genus Rosa and clarifying issues related to phylogeny performed by Russian scientists (Schanzer, 2013, 2015).

The objective of this work is to study the genetic relationships among Rosa accessions of various origins from the NBG’s collection using IRAP and ISSR multilocus markers.

## Materials and methods

As plant material for genotyping, 26 accessions of the genus Rosa L. were selected in the study, 18 of which were cultivated varieties, and 8 belonged to wild species (Table 1). The sample of species included representatives of two subgenera Rosa and Platyrhodon. The subgenus Platyrhodon was represented by a single specimen of the species R. roxburghii Tratt. Cultivated roses were represented by hybrid tea, floribunda, and grandiflora groups of varieties. DNA was extracted from young leaves by the CTAB method (Murray, Thompson, 1980).

**Table 1.**
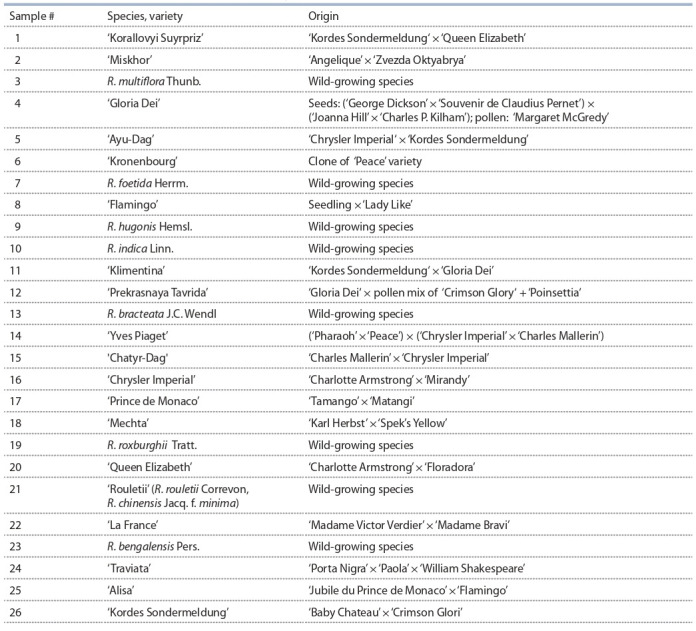
Varieties of the genus Rosa selected for genotyping

ISSR and IRAP markers from various literature sources were chosen for DNA genotyping (Arzate-Fernandez et al., 2005; Jawdat et al., 2010; Krishna Parvathaneni et al., 2011; Yuying et al., 2011; Senkova et al., 2013; Suprun et al., 2014). A total of 32 ISSR markers and 13 IRAP markers were used. The markers were tested for the applicability to genotyping samples of the genus Rosa. The PCR schedule was as follows: predenaturation at 95 °C for 3 min; 35 cycles: denaturation at 95 °C for 35 s, annealing of primers at 50 °C (55 °C in case of IRAP markers) for 1 min, elongation at 72 °C for 1.5 min; postextension at 72 °C for 5 min. Concentrations of reagents in the PCR mixture: 2.5 μl of 10-fold buffer for Taq DNA polymerase (Sibenzyme, Russia), 0.5 or 2.5 μl of dNTP (2.5 mM), 1 unit of Taq DNA polymerase, 2 μl of primer (3.75 mM) and 40–50 ng of total DNA in the total volume of 25 μl. Electrophoresis of PCR products was performed at 100 V in 2.5 % agarose gel stained with ethidium bromide (2 % agarose gel was used for testing markers at 120 V). DNA was visualized under ultraviolet illumination.

On the base of the genotyping results, a binary matrix was constructed for further use of data in statistical processing programs. For statistical processing of the results of ISSR and IRAP genotyping and analysis of genetic relationships of the studied gene pool, the program PAST version 2.17c (UPGMA and PCoA analysis) was used. Structure 2.3.4 (Bayesian analysis) was used to evaluate the genetic structure of the series. Various values of hypothetical populations from K = 2 to K = 7 (burn-in period = 200,000; 500,000 iterations) were used in the calculation.

## Results

**Fig. 1.**
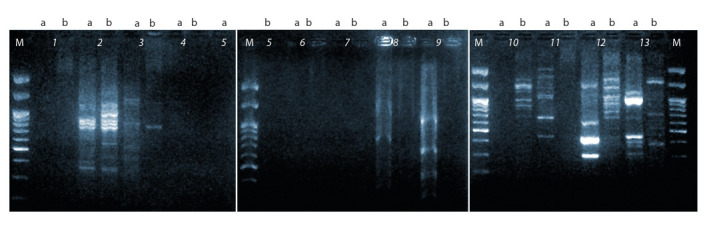
DNA fingerprints of varieties (a) ‘La France’ and (b) ‘Kronenbourg’ tested with IRAP markers. Lane pairs: 1, TDK 1F; 2, TDK 2R; 3, TDK 2F; 4, TDK 12F; 5, TDK 12R; 6, TDK 13F; 7, MET 2F; 8, MET 2R; 9, BARE 1; 10, LTR 3; 11, LTR 15; 12, Cass1; 13, Cass2; M, DNA ladder.

The main criterion for choosing DNA markers was the quality of fingerprints of the tested markers on the rose genotypes (for testing markers, DNA of the varieties ‘La France’ and ‘Kronenbourg’ was used). The spectra of amplified fragments from the studied markers are shown in Fig. 1.

Markers with the best fingerprints were selected for further work. The quality criteria for fingerprints included the number of DNA fragments, their clarity, and brightness in the electrophoretic image. This is necessary for reliable evaluation of genotyping results. Five ISSR (UBC 824, ASSR29, 3A21, UBC 864, and UBC 843), and three IRAP (TDK 2R, Cass1, and Cass2) markers were chosen for genotyping.

A series of 26 genotypes of representatives of the Rosa genus was analyzed with 5 ISSR and 3 IRAP markers (Table 2). For various markers, the ranges of polymorphic alleles varied from 12 to 31 fragments, 19.25 fragments per marker on the average. The UBC 864 marker had the largest number of polymorphic fragments (31), and significant numbers of polymorphic fragments were also found with TDK 2R and ASSR29. Two markers from the set (UBC 864 and UBC 843) gave unique fingerprints for each accession. The UBC 864 marker also had the largest number of unique fragments identified in a single instance in one of the accessions of the sample. Based on the results of genotyping, a binary matrix was constructed for further use of data in statistical processing programs.

**Table 2.**
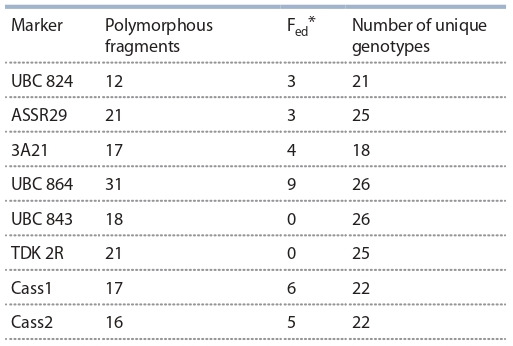
Characteristics of the chosen markers * F_ed_ – the number of unique fragments detected in only one genotype.

**Fig. 2.**
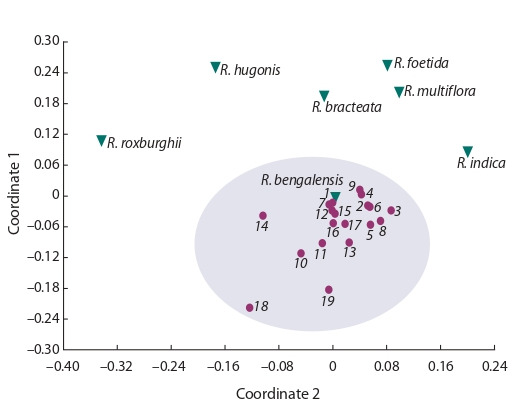
The results of PCoA analysis for studied rose accessions. Dots represent rose cultivars, inverted triangles indicate species accessions. 1, ‘Korallovyi Syurpriz’; 2, ‘Miskhor’; 3, ‘Gloria Dei’; 4, ‘Ayu-Dag’; 5, ‘Kronenbourg’; 6, ‘Flamingo’; 7, ‘Klimentina’; 8, ‘Prekrasnaya Tavrida’; 9, ‘Yves Piaget’; 10, ‘Chatyr- Dag’; 11, ‘Chrysler Imperial’; 12, ‘Prince de Monaco’; 13, ‘Mechta’; 14, ‘Queen Elizabeth’; 15, ‘Rouletii’; 16, ‘La France’; 17, ‘Traviata’; 18, ‘Alisa’; 19, ‘Kordes Sondermeldung’.

Eight markers used allowed us to obtain 153 polymorphic DNA fragments from a series of 26 accessions. This number is sufficient for phylogenetic analysis. Application of the method of principal coordinates (PCoA) to all accessions identified a separate group, including cultivated varieties of roses (Fig. 2). It should be noted that the species R. bengalensis was considered varietal. Among the rose species, the farthest position is occupied by the genotype of R. roxburghii. The species R. hugonis and R. foetida are located at a distance from the bulk of genotypes in the series. The species most closely related to domestic varieties are R. bracteata, R. multiflora, and R. indica. However, R. indica is located separately with regard to the two above-listed species, and its position is closer to domestic forms.

**Fig. 3.**
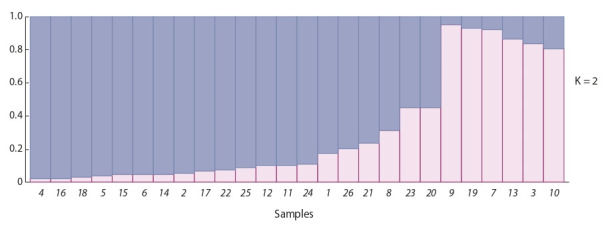
Bar plots of Bayesian analysis in Structure 2.3.4. 1, ‘Korallovyi Syurpriz’; 2, ‘Miskhor’; 3, R. multif lora; 4, ‘Gloria Dei’; 5, ‘Ayu-Dag’; 6, ‘Kronenbourg’; 7, R. foetida; 8, ‘Flamingo’; 9, R. hugonis; 10, R. indica; 11, ‘Klimentina’; 12, ‘Prekrasnaya Tavrida’; 13, R. bracteata; 14, ‘Yves Piaget’; 15, ‘Chatyr-Dag’; 16, ‘Chrysler Imperial’; 17, ‘Prince de Monaco’; 18, ‘Mechta’; 19, R. roxburghii; 20, ‘Queen Elizabeth’; 21, ‘Rouletii’; 22, ‘La France’; 23, R. bengalensis; 24, ‘Traviata’; 25, ‘Alisa’; 26, ‘Kordes Sondermeldung’.

The Bayesian analysis of the results of genotyping accessions with K values ranging from 2 to 7 was performed using Structure 2.3.4 program. At K = 2, accessions of R. multiflora, R. foetida, R. hugonis, R. indica, R. bracteata, and R. roxburghii were allocated to a separate group. The second group was formed by domestic varieties (Fig. 3). The accessions of R. bengalensis, ‘Queen Elizabeth’, and ‘Flamingo’ occupied an intermediate position between these groups. At further increase of K, the trend towards this distribution persists. Within these groups, differentiation by this method is poorly visible. Thus, the method allowed us to reliably divide the series into two major groups: wild rose species and domestic forms.

**Fig. 4.**
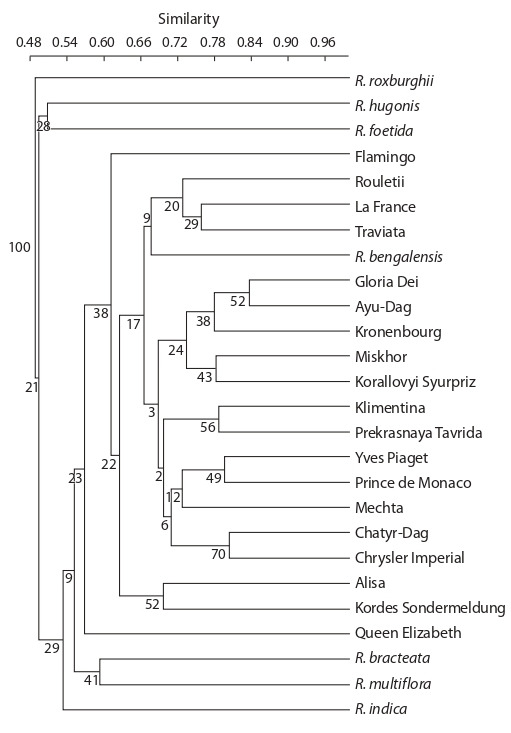
UPGMA clustering results.

The results of clustering by the UPGMA method revealed patterns in the distribution of the studied genotypes, which were also noted when using the PCoA analysis (Fig. 4). In general, the clades of the constructed dendrogram show low bootstrap values. The most remote genotype is R. roxburghii. Two genotypes representing the species R. hugonis and R. foetida form a separate cluster. The next cluster is represented by R. bracteata and R. multiflora. The species R. indica occupies a separate position relative to other genotypes. All species accessions except for R. bengalensis occupy an external position relative to the cluster that includes cultivated forms of roses. Among the rose varieties, the most remote from the total mass of genotypes are ‘Queen Elizabeth’ and ‘Flamingo’. Also, a remote position is occupied by the group represented by varieties ‘Kordes Sondermeldung’ and ‘Alisa’. Other varieties can be divided into 4 clusters: (1) ‘Rouletii’, ‘La France’, ‘Traviata’, and R. bengalensis; (2) ‘Korallovyi Syurpriz’, ‘Ayu-Dag’, ‘Gloria Dei’, ‘Kronenbourg’, and ‘Miskhor’; (3) ‘Chrysler Imperial’, ‘Chatyr-Dag’, ‘Prince de Monaco’, ‘Yves Piaget’, and ‘Mechta’; and (4) ‘Klimentina’ and ‘Prekrasnaya Tavrida’.

## Discussion

In this work, we evaluated the genetic relationship of varieties from the gene pool collection of roses of the Nikita Botanical Gardens. The interpretation of the sample distribution in clustering using various methods is described below.

The Bayesian analysis allows us to determine the genetic contribution of ancestral forms for each genotype studied. Since most rose varieties have a hybrid origin, the analysis of the genetic relationship of samples found that diploid species are clearly separated from cultivated varieties, without making a significant contribution to the gene pool of the studied varieties. However, two varieties have a minor contribution of wild species ‘Flamingo’ and ‘Queen Elizabeth’, in its turn, the genotype of R. bengalensis occupies an intermediate position between the groups of wild species and cultivated varieties. This distribution stems from the origin of cultivated roses from few wild rose species, with one of these species being R. bengalensis.

The isolation of domestic rose forms in clustering can be clearly shown by other methods, such as PCoA and UPGMA. However, in contrast to Bayesian analysis, the other two methods provide a more detailed picture. The PCoA method differentiates wild species by their distance from domestic forms. Similar data on the distribution of samples were obtained by clustering using the UPGMA method. In turn, the low confidence values of the dendrogram clade may be due to the complex hybrid origin of both species and varietal accessions (Bruneau et al., 2007). Therefore, the UPGMA results can be further interpreted when compared with similar data obtained by other methods.

Summarizing the data on the distribution of species samples and cultivated varieties of roses, we can make certain inferences. The phylogenetic data obtained from the results of IRAP and ISSR genotyping are consistent with the information about the systematic position of the studied accessions. The species most distant from the cultivated forms is R. roxburghii, representing the subgenus Platyrhodon of the genus Rosa, and the other species and domestic varieties belong to the subgenus Rosa of the same genus. This distribution agrees with taxonomy data. However, the results of a number of molecular studies on the phylogeny of the genus Rosa do not distinguish the species R. roxburghii from the group of species of the subgenus Rosa (Wissemann, Ritz, 2005; Koopman et al., 2008; Fougère-Danezan et al., 2015). Within the Rosa subgenus, two species of the section Pimpinellifoliae occupy a separate position. Studies conducted on chloroplast DNA markers confirm the proximity of these species (Wissemann, Ritz, 2005). This section of the Rosa subgenus is probably the least close to the cultivated varieties in the series. The types of R. multiflora, R. bracteata, R. indica form the closest clusters with varietal accessions, and their contribution to the formation of the domestic gene pool of roses is not ruled out. Rosa bengalensis forms a common genetic group with domestic varieties. The contribution of this species to the formation of cultivars is beyond question. It should also be noted that the genetic unity of cultivars indicates the generality of their gene pool.

The analysis of genetic relationships among rose varieties from the results of IRAP and ISSR genotyping was based on PCoA and UPGMA clustering data. Comparing the data of these two methods, one can identify the most reliable groups of varieties that are consistently detected by both methods. The first group is ‘Rouletii’ and ‘La France’. Both varieties are of Western European origin, and their relationship is not obvious. The second group is ‘Prince De Monaco’, ‘Yves Piaget’, ‘Chatyr-Dag’, and ‘Chrysler Imperial’. Two varieties from this group have ‘Chrysler Imperial’ among their ancestors: ‘Yves Piaget’ and ‘Chatyr-Dag’. Whereas the relationship of the three varieties ‘Chrysler Imperial’, ‘Yves Piaget’, and ‘Chatyr-Dag’ can be explained by the origin of the last two from the first, their clustering into one group with ‘Prince De Monaco’ is difficult to explain. The third group includes ‘Gloria Dei’, ‘Kronenbourg’, ‘Ayu-Dag’, and ‘Korallovyi Syurpriz’. There are two varieties in this group whose parents have ‘Kordes Sondermeldung’, namely, one variety ‘Gloria Dei’ and its sport (clonal mutant) ‘Kronenbourg’.

Varieties such as ‘Flamingo’, ‘Queen Elizabeth’, ‘Kordes Sondermeldung’ and ‘Alisa’ are the most genetically contrasting However, a number of varieties in the series are derived from these three, such as ‘Ayu-Dag’, ‘Klimentina’, ‘Korallovyi Syurpriz’ (‘Kordes Sondermeldung’), ‘Korallovyi Syurpriz’ (‘Queen Elizabeth’) and ‘Alisa’ (‘Flamingo’).

## Conclusion

The distribution of species accessions of roses in clusters based on their genetic relationships is consistent with the generally recognized phylogeny. It is worth to note that the accession of the species R. bengalensis is included in the cluster formed by cultivated varieties. This fact may point to a contribution of wild roses of Indian origin to the formation of the gene pool of modern rose varieties. In turn, the analysis of the relationship of cultivated varieties of garden roses reveals that the varieties ‘Flamingo’, ‘Queen Elizabeth’, ‘Kordes Sondermeldung’, and ‘Alisa’ stand out from the total mass of the studied varieties. The remaining rose varieties were divided into groups with the greatest genetic similarity. Most of the results of cultivar clustering were explained based on information about the pedigree of varieties, but the position of some varieties was difficult to interpret. Further research is required to determine their relationships. Thus, the markers used in this work have shown their effectiveness in the study of the genus Rosa.

## Conflict of interest

The authors declare no conflict of interest.
